# The prohibitin-binding compound fluorizoline inhibits mitophagy in cancer cells

**DOI:** 10.1038/s41389-021-00352-9

**Published:** 2021-09-27

**Authors:** Sonia Núñez-Vázquez, José Saura-Esteller, Ismael Sánchez-Vera, Emma Guilbaud, Ana M. Cosialls, Gabriel Pons, Jean-Ehrland Ricci, Daniel Iglesias-Serret, Sandrine Marchetti, Joan Gil

**Affiliations:** 1 Departament de Ciències Fisiològiques, Facultat de Medicina i Ciències de la Salut, Universitat de Barcelona, Oncobell-IDIBELL (Institut d’Investigació Biomèdica de Bellvitge), L’Hospitalet de Llobregat, Barcelona, Spain; 2 Université Côte d’Azur, INSERM, C3M Nice, France; 3 Equipe labellisée Ligue Contre le Cancer, Nice, France; 4 Facultat de Medicina, Universitat de Vic–Universitat Central de Catalunya (UVic- UCC), Vic, Barcelona, Spain

**Keywords:** Autophagy, Cancer, Drug discovery

## Abstract

Fluorizoline is a prohibitin-binding compound that triggers apoptosis in several cell lines from murine and human origin, as well as in primary cells from hematologic malignancies by inducing the integrated stress response and ER stress. Recently, it was described that PHB (Prohibitin) 1 and 2 are crucial mitophagy receptors involved in mediating the autophagic degradation of mitochondria. We measured mitophagy in HeLa cells expressing Parkin and in A549, a lung cancer cell line that can undergo mitophagy in a Parkin-independent manner, and we demonstrated that both fluorizoline and rocaglamide A, another PHB-binding molecule, inhibit CCCP- and OA-induced mitophagy. Moreover, we demonstrated that PHBs are mediating Parkin-dependent mitophagy. In conclusion, besides being a potent pro-apoptotic compound, we present fluorizoline as a promising new mitophagy modulator that could be used as anticancer agent.

## Introduction

Mitochondria are crucial for multiple intracellular processes. These organelles are responsible for the generation of ATP through oxidative phosphorylation (OXPHOS) but they also control the intracellular calcium levels, regulate the innate immune response and control the cell death through the initiation of the intrinsic apoptotic pathway [1]. Thus, the selective removal of damaged or dysfunctional mitochondrial is crucial to maintain a proper cell homeostasis [2]. Mitophagy is the form of macroautophagy that ensures the elimination of damaged mitochondria by the lysosomes to maintain the integrity of the mitochondrial pool [3]. Diverse stimuli, including nutrient starvation, higher mitochondrial respiration activity, hypoxia, respiratory chain inhibitors, or iron deficiency can promote mitophagy as a protective mechanism against mitochondrial stress [4].

The best-described mitophagy pathway is driven by PINK1 (PTEN induced putative kinase 1) and Parkin, a RBR (RING-Between-RING) E3 ubiquitin (Ub) ligase. Under basal conditions, mitochondrial transmembrane potential allows the import and processing of PINK1 in the mitochondrial matrix by the protease PARL (Presenilins-associated rhomboid-like protein), leading to its constant degradation. Upon mitochondrial depolarization, PARL cannot longer process PINK1, which leads to its dimerization and autophosphorylation. Once activated and stabilized in the outer mitochondrial membrane (OMM), PINK1 enhances the activation and recruitment of Parkin, which results in the polyubiquitination of numerous OMM proteins that are recognized by the autophagy cargo adaptor p62 and OPTN [5].

Although the PINK1/Parkin signaling pathway is the mechanism of mitophagy most studied and well-characterized, other E3 Ub ligases, such as ARIH1, MUL1, SIAH1 SMURF1, and GP78, have been described to cooperate with, or act alternatively, to Parkin activity downstream of PINK1 [6, 7]. Furthermore, mitophagy can also be mediated by Ub-independent receptor pathways. Several mitochondrial membrane proteins (e.g., PHB2, FUNDC1, BNIP3, NIX, BCL2L13, and FKBP8) and lipids (e.g., cardiolipin and ceramides) can act as mitophagy receptors [8].

Fluorizoline is a fluorinated thiazoline pro-apoptotic compound that induces apoptosis, in a p53-independent manner, in a wide range of cancer cell lines [9]. Moreover, fluorizoline also showed high apoptotic capacity in acute myeloid leukemia [10], chronic lymphocytic leukemia [11], and multiple myeloma [12] primary cancer cells. Fluorizoline directly binds and targets Prohibitin (PHB) 1 and 2 [9, 13], evolutionary conserved and ubiquitously expressed proteins, mainly localized in the inner mitochondrial membrane (IMM), where they interact with each other to form a macromolecular structure [14]. These PHB heteromeric ring-like complexes in the IMM participate in numerous processes in the mitochondria, including protein-quality control, OXPHOS chain synthesis and assembly, ROS formation or mitochondrial DNA organization [14, 15]. In addition, PHB2 was discovered as a novel mitophagy receptor involved in targeting mitochondria for autophagic degradation in mammals [16].

It was described that upon mitochondrial depolarization, PHB2 or PHB2/PHB1 complex directly binds to autophagosome-associated protein LC3 [16]. Furthermore, recently it was further described that upon mitochondrial depolarization, PHB2 binds to PARL, releasing PGAM5 in the process [17], which is responsible for retaining and stabilizing PINK1 on the OMM to initiate mitophagy [18]. Also, it was reported that PHB2 is required for cholestasis-induced mitophagy, where PHB2 brings LC3 to the damaged mitochondria by interacting with p62 and LC3 [19].

The impact of mitophagy on the cellular fate is controversial as the clearance of mitochondria can have diverse effects on tumor development, growth, and progression. Mitophagy can facilitate survival through the adaptation to stress by removing mitochondria that could potentially be permeabilized to induce cell death or, conversely, it can induce cell death due to the excessive removal of mitochondria [20]. On one hand, genetic inhibition of mitophagy pathways sensitizes cancer cells to apoptosis in response to anticancer treatments, supporting the pro-survival role of mitophagy in cancer cells [2]. In fact, enhanced mitophagy contributes to cisplatin and etoposide resistance in cancer cells [21, 22], while mitophagy impairment resensitizes drug-resistant cancer cells [22, 23]. On the other hand, dysfunctional mitochondria and increased mitochondrial ROS can promote tumorigenesis, cancer progression, metastasis, and drug resistance through DNA, lipid, and protein oxidation [2].

Nevertheless, there are several studies suggesting that mitophagy inhibitors in combination with conventional cancer treatment can markedly improve the effectiveness of chemotherapy [24]. For that reason, in this study, we investigated whether fluorizoline can modulate mitophagy by targeting PHBs.

## Material and methods

### Cell culture

HeLa and A549 were supplied by the European Collection of Cell Culture (ECACC). Cells were cultured in Dulbecco’s Modified Eagle Medium (DMEM) supplemented with 10% fetal bovine serum, 100 U/mL penicillin, and 100 ng/mL streptomycin. Cells were cultured at 37 °C in a humidified atmosphere containing 5% carbon dioxide. Cell cultures were periodically tested for mycoplasma contamination by PCR.

### Mito-mKeima mitophagy analysis

HeLa and A549 cells were infected with mito-mKeima (m-Keima) expressing lentiviral vector. The fluorescence profile of this biomarker is pH-dependent, making it a perfect biosensor of mitochondrial degradation by the lysosomes [25]. Excitation at 488 nm and emission at >620 nm was used to detect m-Keima in mitochondria in the cytosol and excitation at 561 nm and emission at >620 nm was used to detect mitochondria in lysosomes (Supplementary Fig. 1A). m-Keima was analyzed by flow cytometry using MACSQuant VYB and MACSQuant software (Miltenyi Biotec, Bergisch Gladbach, Germany).

To calculate the percentage of mitophagy positive cells, 10,000 single events were acquired for each sample and subsequently gated the cells experiencing a shift to acidic m-Keima (Supplementary Fig. 1B).

### Short hairpin RNA

To generate the inducible *PHB2* knockdown in HeLa and A549 cells, lentiviral particles were generated by transfecting HEK 293T cells with the Tet-pLKO-puro vector from Addgene (Watertown, Massachusetts, USA) containing the *PHB2* short hairpin sequence 5′-GACAGAGAGGGCCAAGGACCTCGAGGTCCTTGGCCCTCTCTGTC-3′ under doxycycline promoter. Furtherly, HeLa and A549 cells were infected with these viral particles and selected with 2 μg/mL puromycin.

### Reagents

The synthesis of fluorizoline was performed as previously described [9]. Q-VD-OPh and bafilomycin A were from R&D systems (Minneapolis, Minnesota, USA). CCCP, Oligomycin, and Antimycin A were obtained from Sigma-Aldrich (St Louis, MO, USA). Rocaglamide A was purchased from Enzo Life Sciences (Farmingdale, New York, USA).

### Cell viability

Cell viability was measured by measuring phosphatidylserine exposure by annexin V staining and analyzed by flow cytometry using FACSCanto^TM^ and FACSDiva^TM^ software (Becton Dickinson, NJ, USA). Cell viability was expressed as the percentage of annexin V-negative population, which corresponds to the nonapoptotic cells. Cells were incubated with annexin binding buffer and annexin V for 15 min in the dark before analysis.

### Western blot

Whole-cell protein extracts were obtained by lysing cells with Laemmli sample buffer. Protein concentration was measured with the Micro BCA Protein Assay Reagent kit (Pierce, Rockford, Illinois, USA). 20–40 µg of protein extracts were subjected to reducing conditions, loaded onto a polyacrylamide gel, and then transferred to Immobilon-P membranes from Millipore (Billerica, Massachusetts, USA). One hour after blocking the nonspecific binding sites with 5% (w/v) non-fat milk in Tris-buffered saline with Tween® 20, membranes were incubated overnight at 4 °C with the following specific primary antibodies: β-actin (AC-15, Sigma-Aldrich), PHB1 (sc-28259, Santa Cruz, Dallas, TX, USA), PHB2/REA (07–234, Millipore), VDAC1 (sc-8828, Santa Cruz), HSP60 (4870 S, Cell Signaling, Danvers, MA, USA), GRP75 (sc-13967, Santa Cruz), MTCO2 (ab110258, Abcam, Cambridge, UK), PINK1 (6946, Cell Signaling). Antibody binding was detected using a secondary antibody conjugated to horseradish peroxidase, and the enhanced chemiluminescence detection system (Amersham, Little Chalfont, UK). Quantification of band intensities was conducted using Multi Gauge V3.0 software (FujiFilm Corporation). The relative density of each protein and condition was referred to the internal normalization control.

### Immunostaining

Cells were grown on coverslips before treatment. Cells were washed in PBS, fixed with 4% paraformaldehyde in PBS for 20 min, permeabilized with 0.1% TritonX-100 in PBS for 3 min, and blocked in 2% BSA in PBS for 30 min. Primary antibodies -TOM20 (sc-17764, Santa Cruz), PHB2 (ab182139, abcam), LC3 (14600–1-AP, Proteintech, Manchester, UK)- were used at 1:200 overnight at 4 °C, followed by secondary antibodies at 1:1000 and DAPI (0.5 µg/ml) for 1 h at room temperature. Anti-mouse Alexa Fluor 488 and anti-rabbit Alexa Fluor 647 were obtained from Molecular Probes (Invitrogen, Carlsbad, CA, USA). Cells were mounted with Fluoromount G (SouthernBiotech, Birmingham, AL, USA). Images were acquired with a Carl Zeiss model LSM880 confocal microscope and a Apochromat 63×/1.4 Oil M27 objective lens and they were collected using 405, 488, and 561 nm laser lines for excitation and appropriate emission filters. Images were analyzed with Image J Software. Pearson’s coefficient was obtained with JACoP plugin [26].

### Quantitative RT-PCR

Total DNA was isolated from HeLa cells using the ‘Blood & cell culture DNA mini kit’ from QIAGEN (Hilden, Germany) according to the manufacturer’s protocol. The relative DNA expression levels of APP and COX II were obtained by real-time quantitative PCR, using with SYBR green on the ABI Prism 7900 HT Fast Real-Time PCR System. APP (F: 5′-TTTTTGTGTGCTCTCCCAGGTCT-3′, R: 5′-TGGTCACTGGTTGGTTGGC-3′) and COX II (F: 5′-CGTCTGAACTATCCTGCCCG-3, R: 5′-TGGTAAGGGAGGGATCGTTG-3′). PCR data were captured and analyzed using the Sequence Detector software (SDS version 2.2.2; Applied Biosystems).

### Statistical analysis

The results are shown as the mean ± standard error of the mean (SEM) of values obtained in three or more independent experiments. Statistical analysis was performed using the Student’s *t*-test (two-tailed) or ANOVA-Tukey by using GraphPad Prism 6.0c Software Inc (San Diego, CA, USA). Differences were considered significant at *p* values below 0.05 (*p < 0.05; **p < 0.01; ***p < 0.001).

## Results

### Fluorizoline inhibits mitophagy in cancer cells overexpressing Parkin

To study the effect of fluorizoline in Parkin-mediated mitophagy we performed the experiments in HeLa cells stably expressing Parkin. In order to measure mitophagy, the cells stably expressed mito-mKeima fluorophore, a biosensor of mitochondrial degradation by the lysosomes [27]. This cellular marker changes its fluorescence profile depending on the pH, which allows the measurement of m-Keima fluorescence excitation conversion from green (488 nm-cytosolic) to red (561 nm-mitochondrial) using fluorescence-activated cell sorting (FACS) [25] (Supplementary Fig. 1).

**Fig. 1:**
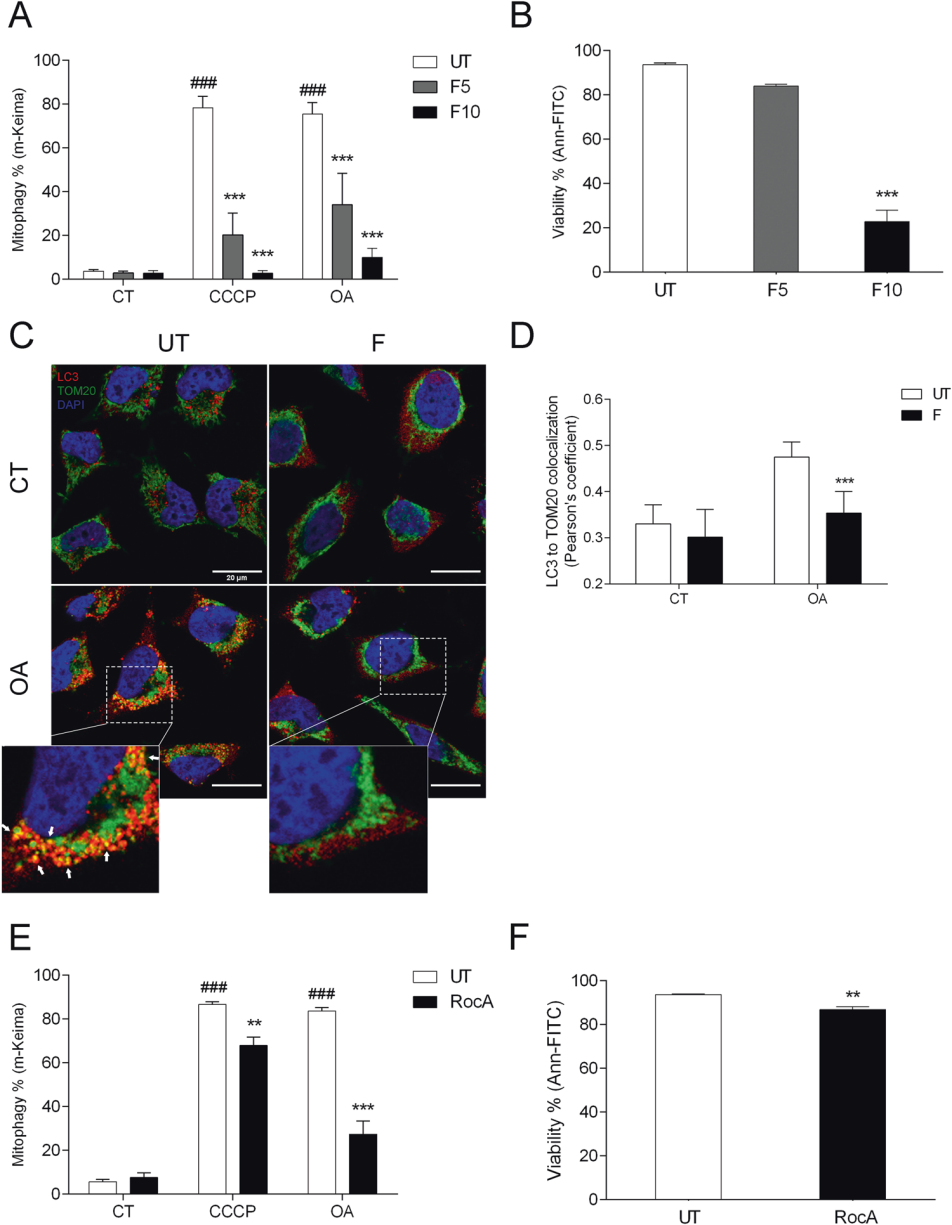
Fluorizoline inhibits mitophagy in cancer cells overexpressing Parkin. HeLa cells overexpressing Parkin (CT) were treated for 16 h with 10 μM CCCP or 1 μM oligomycin/1 μM antimycin A (OA) in the absence (UT) or presence of 5 or 10 μM fluorizoline (F) (**A**, **B**). HeLa Parkin cells treated with 20 μM pan-caspase inhibitor Q-VD-OPh and 40 nM bafilomycin (CT) were treated for 8 h with 1 μM oligomycin/1 μM antimycin (OA), in the absence (UT) or presence of 10 μM fluorizoline (F). Cells were co-immunostained for DAPI (blue), TOM20 (green), and LC3 (red) and co-localization was analyzed by confocal microscopy. White arrows indicate the mitochondrial marker within the autophagosome. These are representative images of three independent experiments (**C**). The co-localization between LC3 and TOM20 was measured and is represented as the Pearson’s coefficient (*n* = 3) (**D**). HeLa cells overexpressing Parkin (CT) were treated for 16 h with 10 μM CCCP or 1 μM oligomycin/1 μM antimycin A (OA), in the absence (UT) or presence of 500 nM rocaglamide A (RocA) (**E**, **F**). m-Keima was measured by flow cytometry and it is expressed as the mean ± SEM (*n* = 5 independent experiments) of the percentage of mitophagy positive cells (**A**, **E**). Viability was measured by flow cytometry and it is expressed as the mean ± SEM (*n* = 4 independent experiments) of the percentage of non-apoptotic cells (annexin V-negative) (**B**, **F**). ^###^*p* < 0.001 CT versus CCCP and OA-treated cells and **p < 0.01 and ***p < 0.001 UT versus F or RocA-treated cells.

We treated HeLa Parkin cells for 16 h with 10 μM protonophore carbonyl cyanide m-chlorophenyl hydrazone (CCCP) or a combination of 1 μM oligomycin and 1 μM antimycin A (OA), to depolarize the mitochondria and induce mitophagy. The results showed that the addition of 5 or 10 μM fluorizoline can block CCCP and OA-induced mitophagy (Fig. 1A). Strikingly, even the lower dose of fluorizoline that does not induce a pronounced cell death can inhibit the mitophagy outcome (Fig. 1B).

Next, we studied the co-localization of LC3 and the mitochondrial marker TOM20 in HeLa Parkin cells upon mitophagy induction by 1 μM OA, in the presence or absence of 10 μM fluorizoline. Cells were treated with 20 μM Q-VD-OPh, a caspase inhibitor to avoid the appearance of apoptotic bodies, and with 40 nM bafilomycin A, an inhibitor of the autophagosome-lysosome fusion that facilitates the accumulation of LC3-II in the cells. We checked that the addition of Q-VD-OPh did not modulate the effects of fluorizoline in the mitophagy process (Supplementary Fig. 2). Upon 8 h of mitophagy induction, we can observe a significant increase in the co-localization of LC3 with TOM20 (Fig. 1C, D). However, when fluorizoline is present this co-localization is lost, demonstrating its inhibitory role in the process.

In parallel, we decided to check whether other PHB binding molecules could also block mitophagy induction; thus, we tested the natural PHB-binding compound rocaglamide A [28]. Measuring the conversion of m-Keima, we observed that 500 nM rocaglamide A is able to block CCCP and OA-induced mitophagy (Fig. 1E). Similar to fluorizoline treatment, rocaglamide A was able to inhibit mitophagy in a dose that does not induce high levels of apoptosis (Fig. 1F).

### Fluorizoline inhibits the removal of damaged mitochondria

To further characterize the ability of fluorizoline to prevent mitophagy we monitored the removal of damaged mitochondria by measuring the disappearance of mitochondrial markers.

**Fig. 2:**
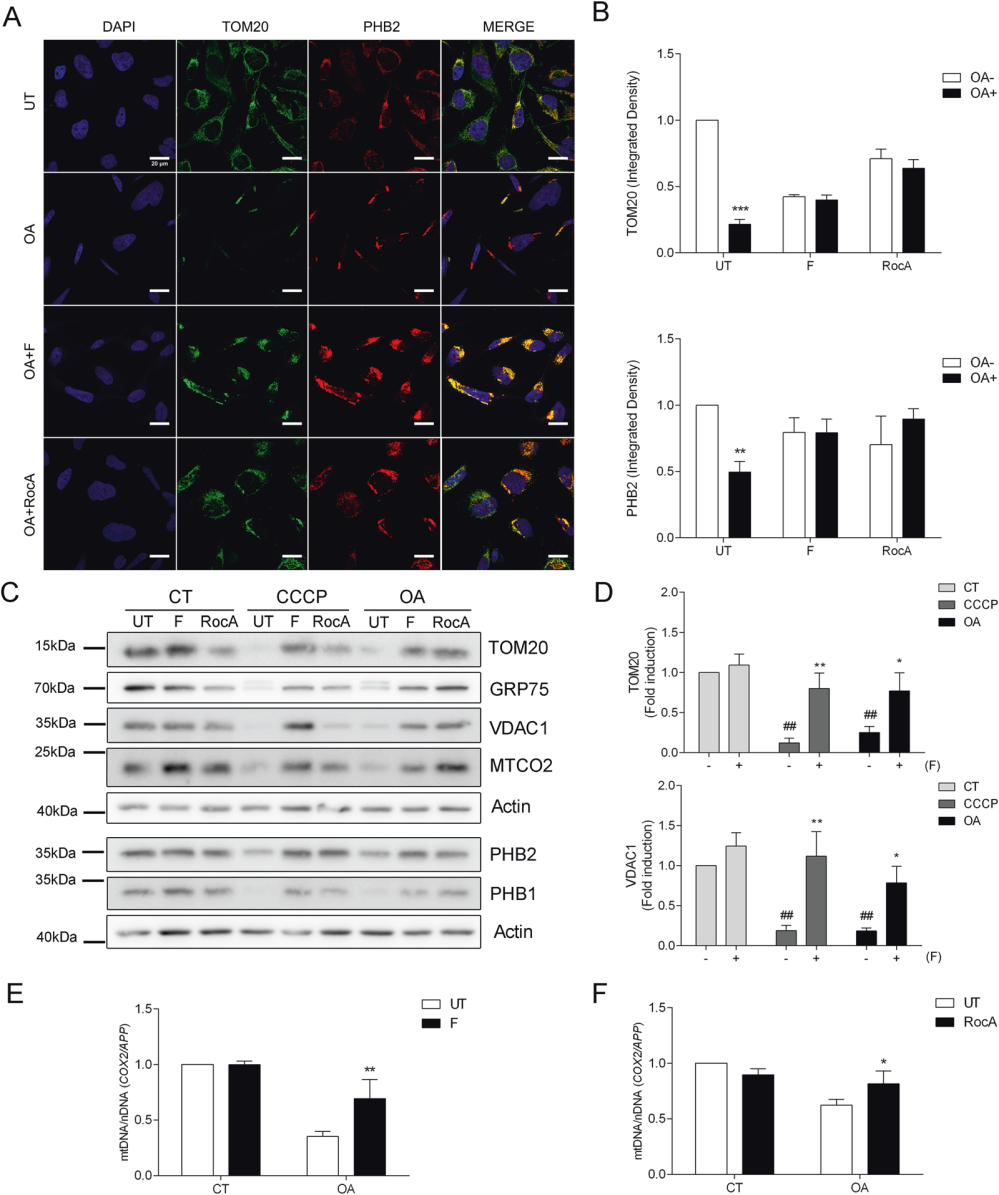
Fluorizoline inhibits the removal of damaged mitochondria. HeLa Parkin cells (CT) were treated with 20 μM pan-caspase inhibitor Q-VD-OPh (**A**, **B**, **E**, **F**) or not (**C**, **D**) while treated for 16 h with 1 μM oligomycin/1 μM antimycin (OA) or 10 μM CCCP, in the absence (UT) or presence of 10 μM fluorizoline (F) or 500 nM rocaglamide A (RocA). Cells were co-immunostained for DAPI (blue), TOM20 (green), and PHB2 (red) and co-localization was analyzed by confocal microscopy (**A**). The integrated density of TOM20 and PHB2 was quantified. Mean ± SEM (*n* = 3 independent experiments) (**B**). Protein levels from whole-cell lysates were analyzed by western blot and actin was used as a loading control (**C**). TOM20 and VDAC1 protein expression levels were quantified. Mean ± SEM (*n* = 3 independent experiments) (**D**). The genes for COX II and the amyloid precursor protein (APP) were amplified and measured by RT-qPCR as indicatives of mitochondrial and nuclear DNA, respectively. Mean ± SEM (*n* = 3 independent experiments) (**E**, **F**). These are representative images of three independent experiments (**A**, **C**). **p < 0.01 and ***p < 0.001 CT versus OA-treated cells (**B**); *p < 0.05, **p < 0.01 UT versus F or RocA-treated cells (**D**−**F**); ^##^*p* < 0.01 CT versus CCCP and OA-treated cells (**D**).

First, we performed an immunofluorescence staining against the mitochondrial proteins TOM20 and PHB2 in HeLa Parkin cells treated with 20 μM Q-VD-OPh. The loss of mitochondria in HeLa Parkin cells upon 1 μM OA treatment was clearly observed as the signal of TOM20 and PHB2 significantly disappeared (Fig. 2A). On the contrary, the addition of 10 μM fluorizoline during OA treatment significantly prevented the loss of TOM20 and PHB2 staining (Fig. 2B and Supplementary Fig. 3). Similar protective effects were observed with 500 nM rocaglamide A (Fig. 2A, B and Supplementary Fig. 3).

Then, we proceed to measure the disappearance of TOM20 and PHB2 by immunoblot, alongside several others mitochondrial markers, such as GRP75, VDAC1, MTCO2, and PHB1. HeLa Parkin cells were treated for 16 h with 10 μM CCCP or 1 μM OA while treated with 10 μM fluorizoline or 500 nM rocaglamide A. We could confirm that both PHB binding compounds block the degradation of mitochondrial proteins induced by CCCP and OA (Fig. 2C, D). The specificity of these proteins to monitor mitophagy was confirmed when HeLa cells lacking Parkin were treated for 16 h with CCCP and OA and did not experience any modulations of these proteins (data not shown).

Next, we measured the removal of damaged mitochondria by measuring the degradation of mitochondrial DNA upon treatment. We chose COX II and amyloid precursor protein (APP) genes as indicatives of mitochondrial and nuclear DNA, respectively [29]. As expected, 10 μM fluorizoline treatment prevented the degradation of mitochondrial DNA induced by 1 μM OA (Fig. 2E), in a similar way that 500 nM rocaglamide A did (Fig. 2F).

Therefore, by measuring the disappearance of mitochondrial proteins and DNA, we can conclude that both PHB binding compounds, fluorizoline and rocaglamide A, inhibit the removal of damaged mitochondria in cancer cells.

### Fluorizoline inhibits Parkin-independent mitophagy

**Fig. 3:**
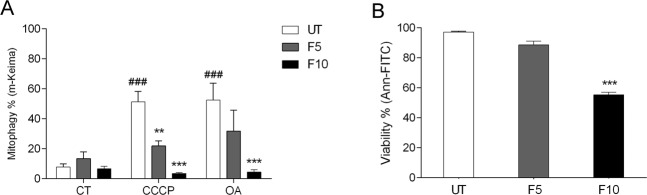
Fluorizoline inhibits Parkin-independent mitophagy. A549 cells (CT) were treated for 24 h with 10 μM CCCP or 1 μM oligomycin/1 μM antimycin A (OA) in the absence (UT) or presence of 5 or 10 μM fluorizoline (F). m-Keima was measured by flow cytometry and it is expressed as the mean ± SEM (*n* = 5 independent experiments) of the percentage of mitophagy positive cells (**A**). Viability was measured by flow cytometry and it is expressed as the mean ± SEM (*n* = 5 independent experiments) of the percentage of non-apoptotic cells (annexin V-negative) (**B**). ^###^*p* < 0.001 CT versus CCCP and OA-treated cells; **p < 0.01 and ***p < 0.001 UT versus F-treated cells.

Next, we studied whether fluorizoline could inhibit Parkin-independent mitophagy. We decided to use A549 lung carcinoma cells, in which mitophagy depends on the endogenous expression of the E3 ligase ARIH1 and does not express Parkin [21]. A549 cells were treated for 24 h with 10 μM CCCP or 1 μM OA in the presence or absence of 5 μM or 10 μM fluorizoline and we measured m-Keima shift. Indeed, the results showed that fluorizoline was also able to inhibit the mitophagy process in this cell line (Fig. 3A).

In addition, compared to HeLa cells, we observed that A549 cells were less sensitive to fluorizoline-induced apoptosis at the same doses (Fig. 3B), but they exhibit similar levels of mitophagy inhibition, which confirms that the inhibition observed is not a consequence of the pro-apoptotic effects of fluorizoline.

### Fluorizoline prevents PINK1 stabilization in the OMM

Recently, it was reported that upon mitochondrial depolarization, PHB2 binds to PARL instead of interacting with PGAM5, therefore allowing PGAM5 to stabilize PINK1 in the OMM, to recruit Parkin, and to initiate the mitophagy process [17]. Therefore, we studied whether fluorizoline treatment decreases PINK1 protein levels.

**Fig. 4:**
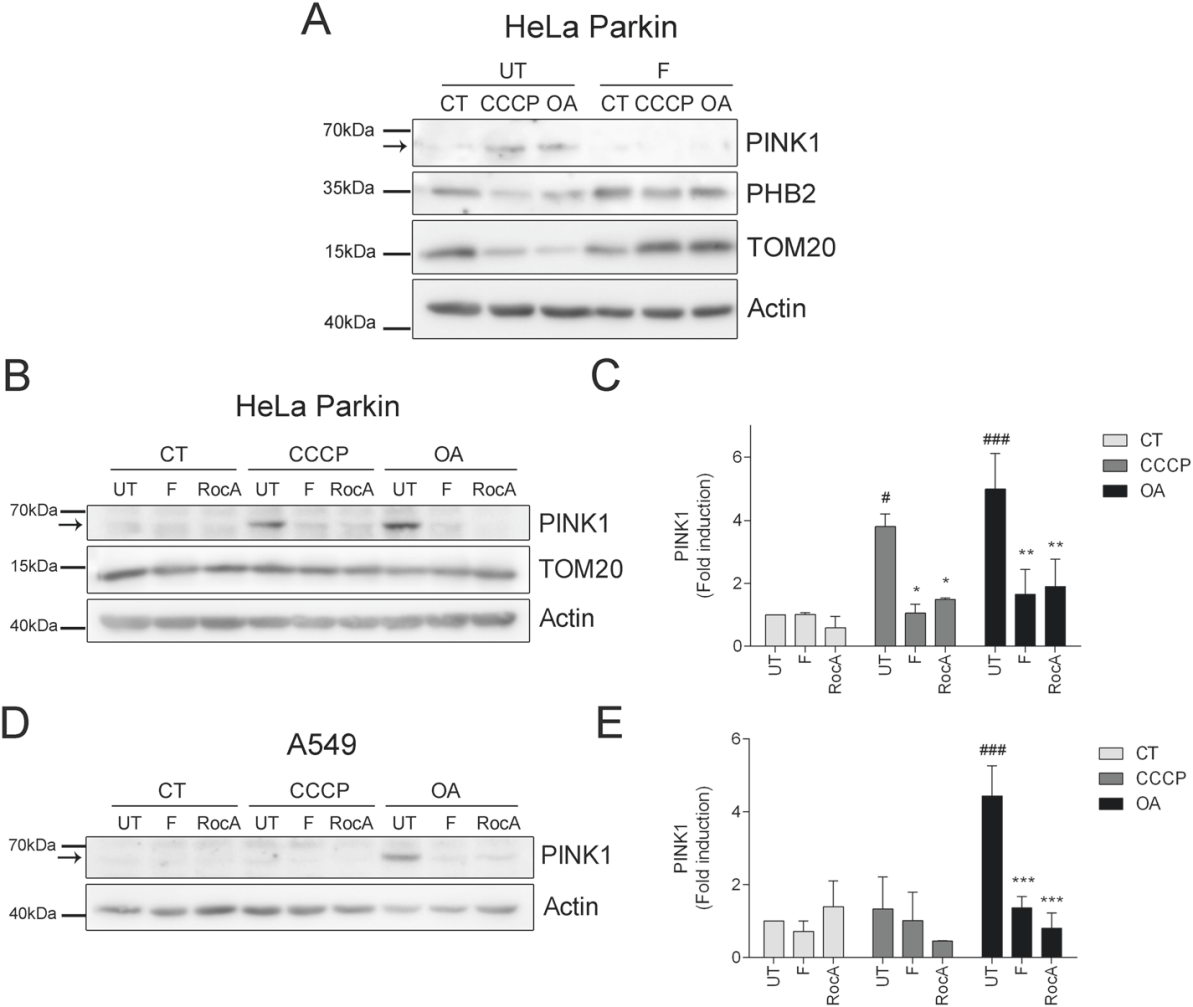
Fluorizoline prevents PINK1 stabilization in the OMM. HeLa Parkin cells (CT) were treated for 16 h with 10 μM CCCP or 1 μM oligomycin/1 μM antimycin A (OA) in the absence (UT) or presence of 10 μM fluorizoline (F) (**A**). HeLa Parkin (**B**, **C**) and A549 (**D**, **E**) cells were treated for 6 h with or without 10 μM CCCP or 1 μM oligomycin/1 μM antimycin A (OA) in the absence (UT) or presence of 10 μM fluorizoline (F) or 500 nM rocaglamide A (RocA). Protein levels from whole-cell lysates were analyzed by western blot and actin was used as loading control. These are representative images of three independent experiments (**A, B, D**). PINK1 protein expression levels were quantified. Mean ± SEM (*n* = 3 independent experiments) (**C, E**). *p < 0.05, **p < 0.01, ***p < 0.001 UT versus F or RocA-treated cells; ^#^*p* < 0.05, ^###^*p* < 0.001 CT versus CCCP and OA-treated cells.

Fluorizoline prevented the accumulation of PINK1 in HeLa Parkin cells induced by a 16 h treatment of 10 μM CCCP and 1 μM OA, accompanied by a stabilization of TOM20 and PHB2 protein levels (Fig. 4A). To confirm that fluorizoline was inhibiting mitophagy initial steps, we checked for PINK1 protein levels after a shorter treatment of CCCP and OA. We were able to observe PINK1 protein stabilization with CCCP and OA in HeLa Parkin cells (Fig. 4B, C) and with OA in A549 cells (Fig. 4D, E) after 6 h of treatment. In both cases, PINK1 protein accumulation was inhibited by 10 μM fluorizoline and 500 nM rocaglamide A. As this process takes place at the beginning of the mitophagy process, TOM20 protein levels still remain stable (Fig. 4B). Thus, we confirmed both fluorizoline and rocaglamide A prevent PINK1 stabilization in the OMM upon mitochondria depolarization.

### Effect of PHB depletion in Parkin-dependent and -independent mitophagy

**Fig. 5:**
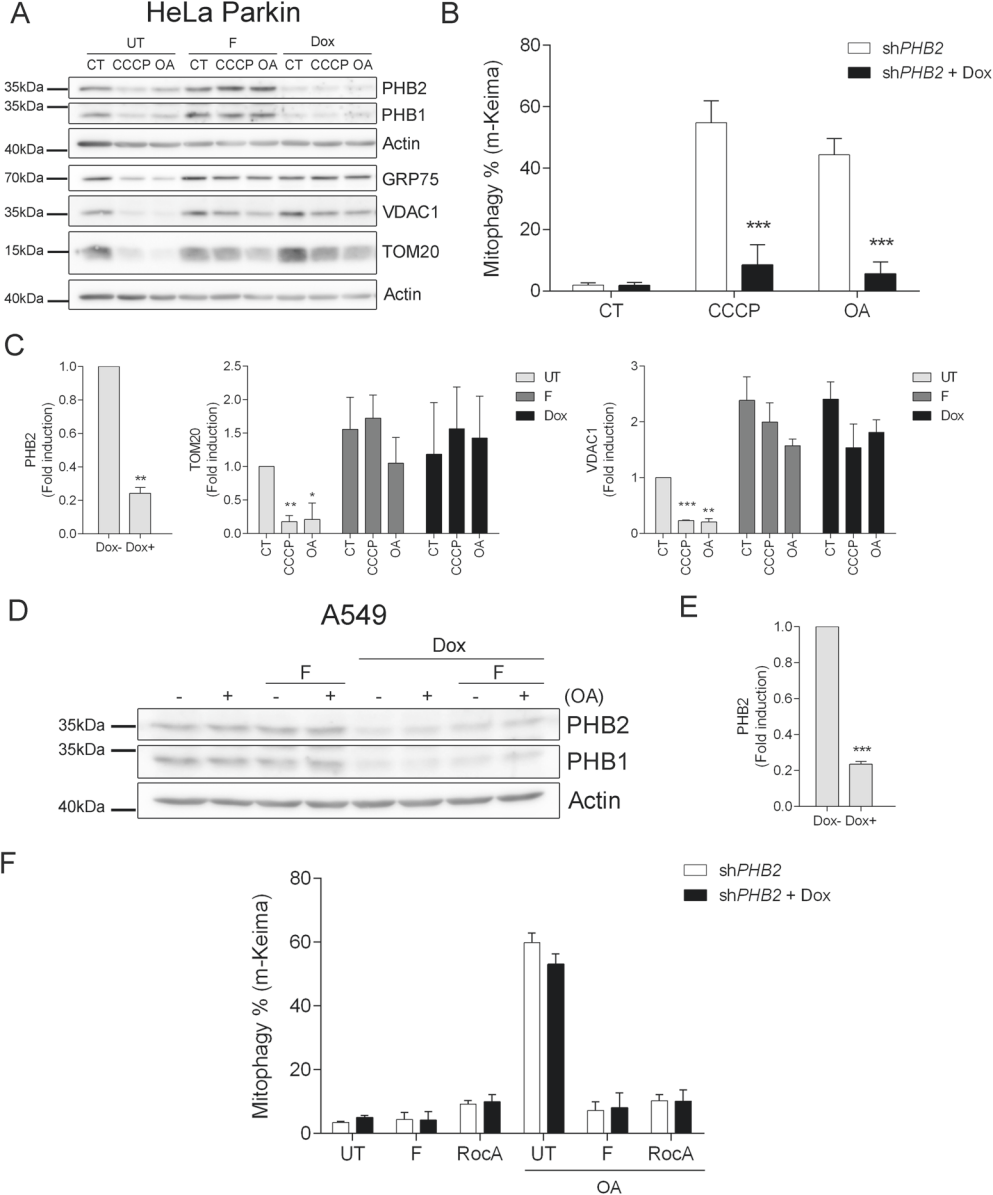
Effect of PHB depletion in Parkin-dependent and -independent mitophagy. HeLa Parkin sh*PHB2* cells (CT) were treated for 16 h with 10 μM CCCP or 1 μM oligomycin/1 μM antimycin A (OA) in the absence (UT) or presence of 10 μM fluorizoline (F), or after a 72 h 200 ng/mL doxycycline (Dox) treatment (**A, C**). HeLa Parkin sh*PHB2* cells (CT) were treated with 200 ng/mL doxycycline for 72 h and then treated with 10 μM CCCP or 1 μM oligomycin/1 μM antimycin A (OA) for 16 h (**B**). A549 sh*PHB2* cells (CT), previously treated or not with 200 ng/mL doxycycline (Dox) for 72 h, were treated with 1 μM oligomycin/1 μM antimycin A (OA) for 24 h in the absence (UT) or presence of 10 μM fluorizoline (F) or 500 nM rocaglamide A (RocA) (**D**, **F**). Protein levels from whole-cell lysates were analyzed by western blot and actin was used as a loading control. These are representative images of three independent experiments (**A**, **D**). m-Keima was measured by flow cytometry and referenced to their corresponding untreated controls and it is expressed as the mean ± SEM (*n* = 3 independent experiments) of the percentage of mitophagy positive cells (**B**, **F**). PHB2, TOM20, and VDAC1 protein expression levels were quantified. Mean ± SEM (*n* = 3 independent experiments) (**C**, **E**). ***p < 0.001 Dox-treated versus Dox-non treated cells (**B**); *p < 0.05, **p < 0.01, ***p < 0.001 CT versus CCCP and OA-treated cells (**C**, **E**).

To further study the role of PHBs in the inhibition of mitophagy by fluorizoline, we infected HeLa Parkin cells with a short hairpin RNA (shRNA) against *PHB2* under the control of an inducible promoter regulated by doxycycline. Upon 72 h treatment with 200 ng/mL doxycycline, the induction of sh*PHB2* decreased the expression of PHB2 and PHB1 (Fig. 5A, C), as both PHBs are interdependent at the protein level [30, 31]. PHB depleted cells, when treated with 10 μM CCCP and 1 μM OA, were unable to undergo mitophagy, similarly to when cells were treated with 10 μM fluorizoline (Fig. 5B). Moreover, the absence of PHBs had a similar effect than fluorizoline treatment when monitoring the removal of damaged mitochondria, as the levels of GRP75, VDAC1, and TOM20 remained stable upon CCCP and OA treatment (Fig. 5A, C).

Since most studies proving PHB2 involvement in mitophagy were reported in cells undergoing mitophagy in a Parkin-dependent manner, and considering that we had demonstrated that fluorizoline could also block Parkin-independent mitophagy, we wanted to check whether PHB2 was also essential to mediate mitophagy in A549 cells. sh*PHB2* A549 cells were treated for 24 h with 1 μM OA, after a previous 72 h treatment with 200 ng/mL doxycycline to delete PHBs (Fig. 5D, E), and m-Keima measurement showed that A549 lacking PHBs could undergo mitophagy (Fig. 5F). Furthermore, both 10 μM fluorizoline and 500 nM rocaglamide A were able to inhibit OA-induced mitophagy in PHB-depleted A549 cells at the same level than in control cells.

## Discussion

In this manuscript, we described that the PHB-binding compound fluorizoline can inhibit Parkin-dependent and -independent mitophagy. Moreover, while the presence of PHB is essential to undergo mitophagy in HeLa cells overexpressing Parkin, A549 cells appear to have alternative mechanisms to induce mitophagy, independently of PHB. Therefore, although fluorizoline was first thought to be inhibiting mitophagy by binding to PHB, the possibility of fluorizoline having other targets is left open. Despite this, we do not fully discard the possibility that PHB2 is in some level involved in the mitophagy process in A549. Rocaglamide A has already been described to inhibit mitophagy by targeting PHBs [17] and, although it is possible, it is very unlikely that fluorizoline and rocaglamide A share another target besides PHB that is also involved in mitophagy. Thus, the role of PHB in Parkin-independent mitophagy should be further analyzed.

Since it was first described by Levine and colleagues that PHB2 is an IMM mitophagy receptor that binds to LC3 upon mitochondrial depolarization [16], several studies have focused on the implication of PHB2 in this process. Recently, it was described that PHB2 promotes PINK1 stabilization in the OMM through the PARL-PGAM5-PINK1 axis [17]. In this study, they also tested the flavagline PHB ligands, FL3 and rocaglamide A, and they observed that those compounds inhibit PINK1/Parkin-mediated mitophagy. Both compounds were able to inhibit the elimination of mitochondria and the recruitment of Parkin and PINK1 at the OMM, at the same time that their treatment caused an increase in PARL protein levels. Based on this work, fluorizoline would prevent the PGAM5 proteolytic protection conferred by PHB and therefore, PARL would constantly process PGAM5, not allowing the stabilization of PINK1.

There are some contradictions in the literature about the role of PHB2 ligands regarding mitophagy. Although rocaglamide A enhances natural killer cell-mediated lysis through inhibition of autophagy [32], another study showed that rocaglamide A can also act as a mitophagy inducer in pancreatic cancer cells [33]. This cell type-dependent role of PHB2 ligands in mitophagy could be related to the dual role of mitophagy in cell death and survival.

Mitophagy and, more generally, autophagy is a pro-survival stress response; however, prolonged over-activation of the autophagosomal/lysosomal pathway can lead to autophagic cell death [34]. A similar threshold is present to define the cellular outcome during the ISR and ER stress [35], both processes involved in the mechanism of fluorizoline-induced apoptosis [36]. While the stress response of these signaling pathways is pro-apoptotic in HeLa and HAP1 cells [36], it has a pro-survival role in HEK293T and U2OS cells [37]. Therefore, we hypothesize that the response to the ISR upon fluorizoline treatment might be coordinated with the inhibition or induction of mitophagy as a global adaptive response to prevent or to promote fluorizoline-induced apoptosis. In this hypothesis, the pro-apoptotic induction of the ISR by fluorizoline in HeLa cells would be accompanied by an inhibition of mitophagy, while the pro-survival activation of ISR in HEK293T would be linked to an induction of mitophagy to prevent the apoptotic outcome of fluorizoline treatment.

Supporting this idea, it was demonstrated that inhibition of autophagy enhances ER stress-induced cisplatin cytotoxicity in HeLa cells [38]. Furthermore, mitophagy induction has been associated with drug resistance and the inhibition of autophagy can sensitize tumor cells to the cytotoxicity of anticancer drugs [39]. In addition, the UPR derived from ER stress can induce mitophagy to clear stress-damaged mitochondria [40].

This opens the possibility to use fluorizoline in combination with other chemotherapeutic treatments to avoid future resistances or to avoid ER stress pro-survival effects of anticancer drugs. There are several studies suggesting that mitophagy inhibitors in combination with conventional cancer treatment can markedly improve the effectiveness of chemotherapy [23, 41, 42]. However, it seems that both mitophagy inducers and inhibitors may be effective in anti-cancer therapy [20]. For instance, dihydroergotamine was shown to induce cell death in A549 cells by inducing mitophagy [43]. Also, mitochondrial fragmentation caused by the anti-cancer drug phenanthroline promotes mitophagy [44]. Moreover, mitophagy stimulated by low-intensity ultrasound therapy in the presence of curcumin induces cell death in nasopharyngeal carcinoma cells [45]. On the contrary, the inhibition of mitophagy can also represent an effective strategy for therapy, as it has been demonstrated that it might enhance the cell death induced by several anticancer drugs. For instance, Drp1-GTPase MDIVI-1 inhibits mitophagy by disrupting the mitochondrial fragmentation process, which helps to overcome tumor cell resistance to cisplatin treatment [23]. Also, the mitophagy inhibitor liensinin sensitizes triple-negative human breast cancer cells to doxorubicin treatment [46].

Taken together, this study showed that pro-apoptotic PHB-binding compound fluorizoline can block the process of mitophagy in cancer cells. Furthermore, it also exposed the need to further analyze the role of mitophagy receptor PHB2 in Parkin-independent mitophagy. In conclusion, besides a potent pro-apoptotic compound, we present fluorizoline as a promising new mitophagy modulator as an anticancer agent.

## Supplementary information

Supplementary figure 1

Supplementary figure 2

Supplementary figure 3

Supplementary figure legends
