# Supplementary figures and images for: The prohibitin-binding compound fluorizoline inhibits mitophagy in cancer cells
Source: Oncogenesis. 2021 Sep 27;10(9):64. doi: 10.1038/s41389-021-00352-9 (PMC8476632; doi:10.1038/s41389-021-00352-9)

Supplementary Figure 1

A

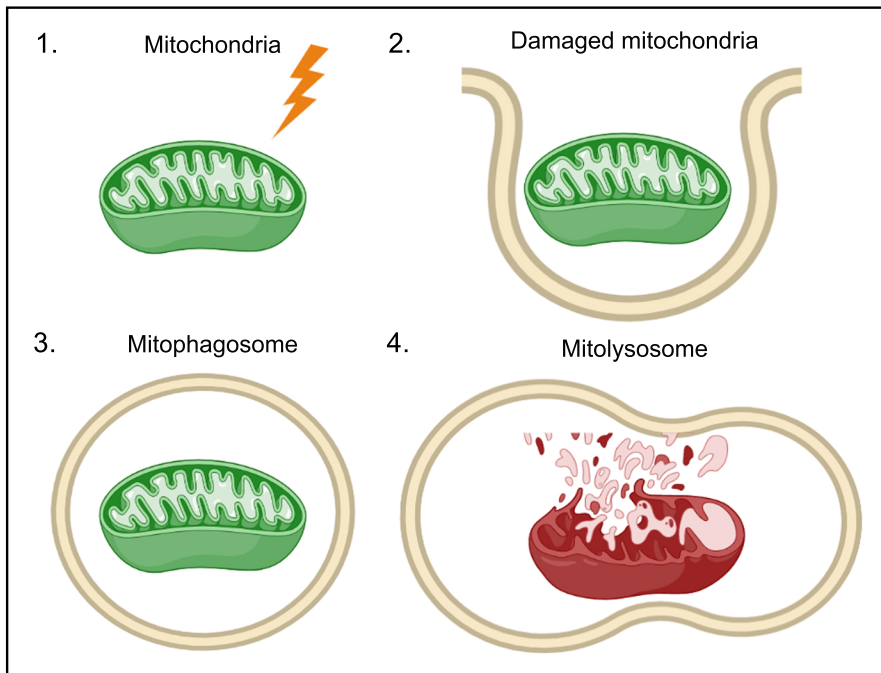

B

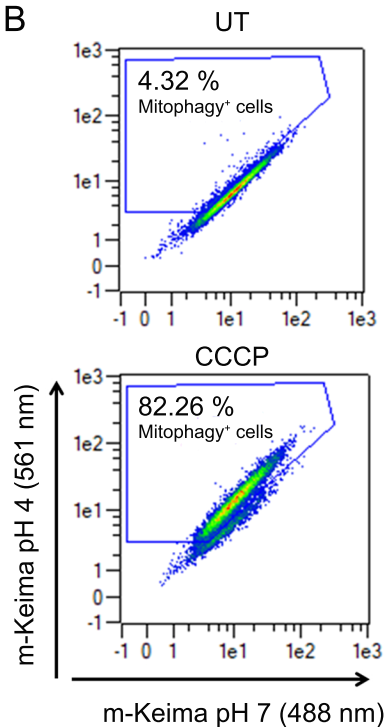

Supplement: Supplementary file 1 — Supplementary figure 1 [file 41389_2021_352_MOESM1_ESM.pdf]

## Supplementary Figure 2

A

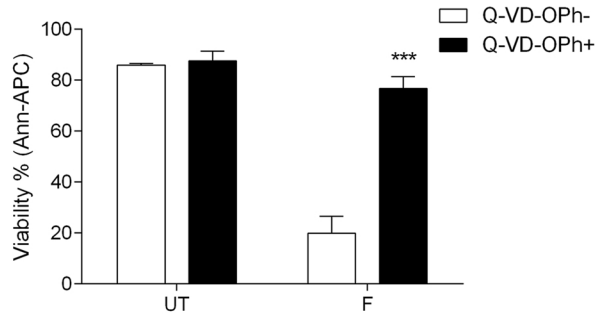

B

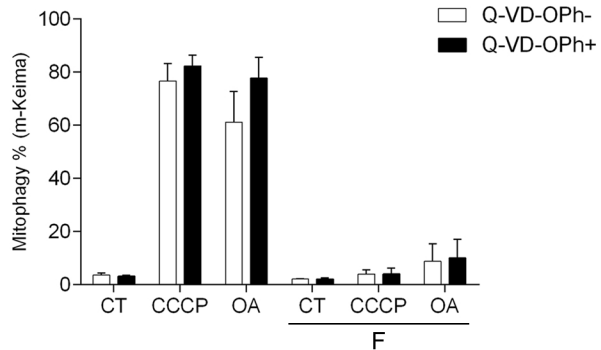

Supplement: Supplementary file 2 — Supplementary figure 2 [file 41389_2021_352_MOESM2_ESM.pdf]

# Supplementary Figure 3

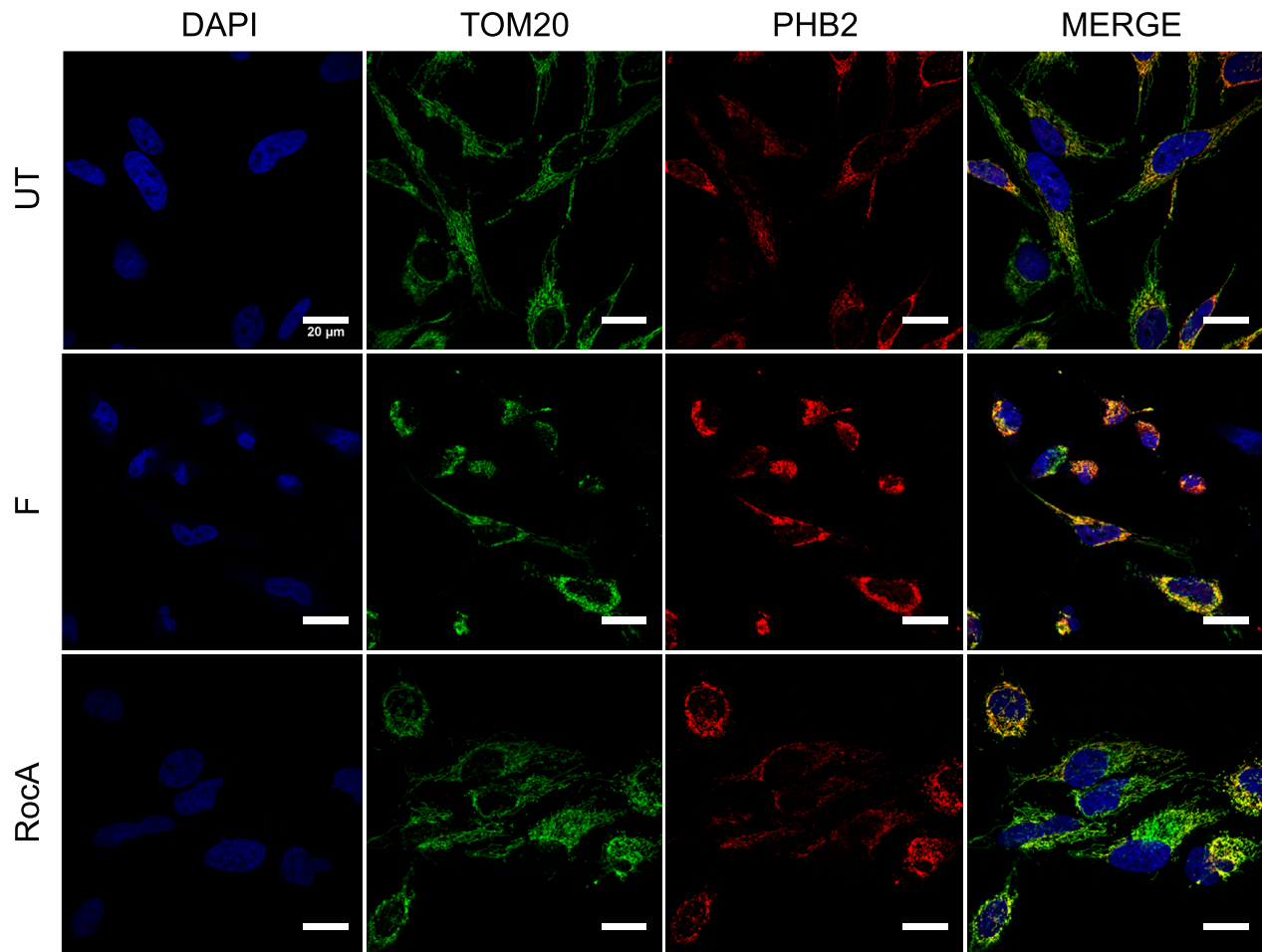

Supplement: Supplementary file 3 — Supplementary figure 3 [file 41389_2021_352_MOESM3_ESM.pdf]
