# Supplementary material for: The prohibitin-binding compound fluorizoline inhibits mitophagy in cancer cells
Source: Oncogenesis. 2021 Sep 27;10(9):64. doi: 10.1038/s41389-021-00352-9 (PMC8476632; doi:10.1038/s41389-021-00352-9)
Supplement: Supplementary file 4 — Supplementary figure legends [file 41389_2021_352_MOESM4_ESM.docx]

**Supplementary Figure 1.** Upon a mitochondrial stress (1), damaged mitochondria are engulfed into phagophores (2), forming mitophagosomes (3) that will fusion with lysosomes, leading to the appearance of mitolysosomes (4). The pH-dependent fluorescence profile of m-Keima allows the measurement of its fluorescence excitation conversion from green (cytosolic) to red (mitochondrial) that occurs during mitophagy **(A)**. HeLa Parkin cells expressing m-Keima were untreated (UT) or treated with 10 μM CCCP for 16 hours and analyzed by fluorescence-activated cell sorting (FACS). Green fluorescence (488 nm) of m-Keima reflects mitochondria in the cytosol (pH 7), while red fluorescence (561 nm) reflects mitochondria in lysosomes (pH 4). The cellular population in the panel represents the percentage of mitophagy^+^ cells. Created with BioRender.com.

**Supplementary figure 2.** HeLa cells were untreated (UT) or treated with 10 μM fluorizoline (F), in the presence or absence of 20 μM pan-caspase inhibitor Q-VD-OPh. Viability was measured by flow cytometry and it is expressed as the mean ± SEM (n=3 independent experiments) of the percentage of non-apoptotic cells (annexin V-negative) **(A)**. HeLa Parkin cells (CT) were treated or not with 20 μM pan-caspase inhibitor Q-VD-OPh while treated for 16 hours with 10 μM CCCP or 1 μM oligomycin/1 μM antimycin A (OA), in the presence or absence of 10 μM fluorizoline (F). m-keima was measured by flow cytometry and it is expressed as the mean ± SEM (n=3 independent experiments) of the percentage of mitophagy positive cells **(B)**. ***p< 0.001 Q-VD-OPh-treated versus Q-VD-OPh-non treated cells.

**Supplementary figure 3.** HeLa Parkin cells were treated with 20 μM pan-caspase inhibitor Q-VD-OPh, while untreated (UT) or treated for 16 hours with 10 μM fluorizoline (F) or 500 nM rocaglamide A (RocA) and co-immunostained for DAPI (blue), TOM20 (green) and PHB2 (red). Co-localization was analyzed by confocal microscopy. These are representative images of at least three independent experiments.
